# Supplementary figures and images for: Sex-specific effects of appetite suppressants on stereotypy in rats
Source: PLoS One. 2025 Jun 24;20(6):e0325067. doi: 10.1371/journal.pone.0325067 (PMC12186957; doi:10.1371/journal.pone.0325067)

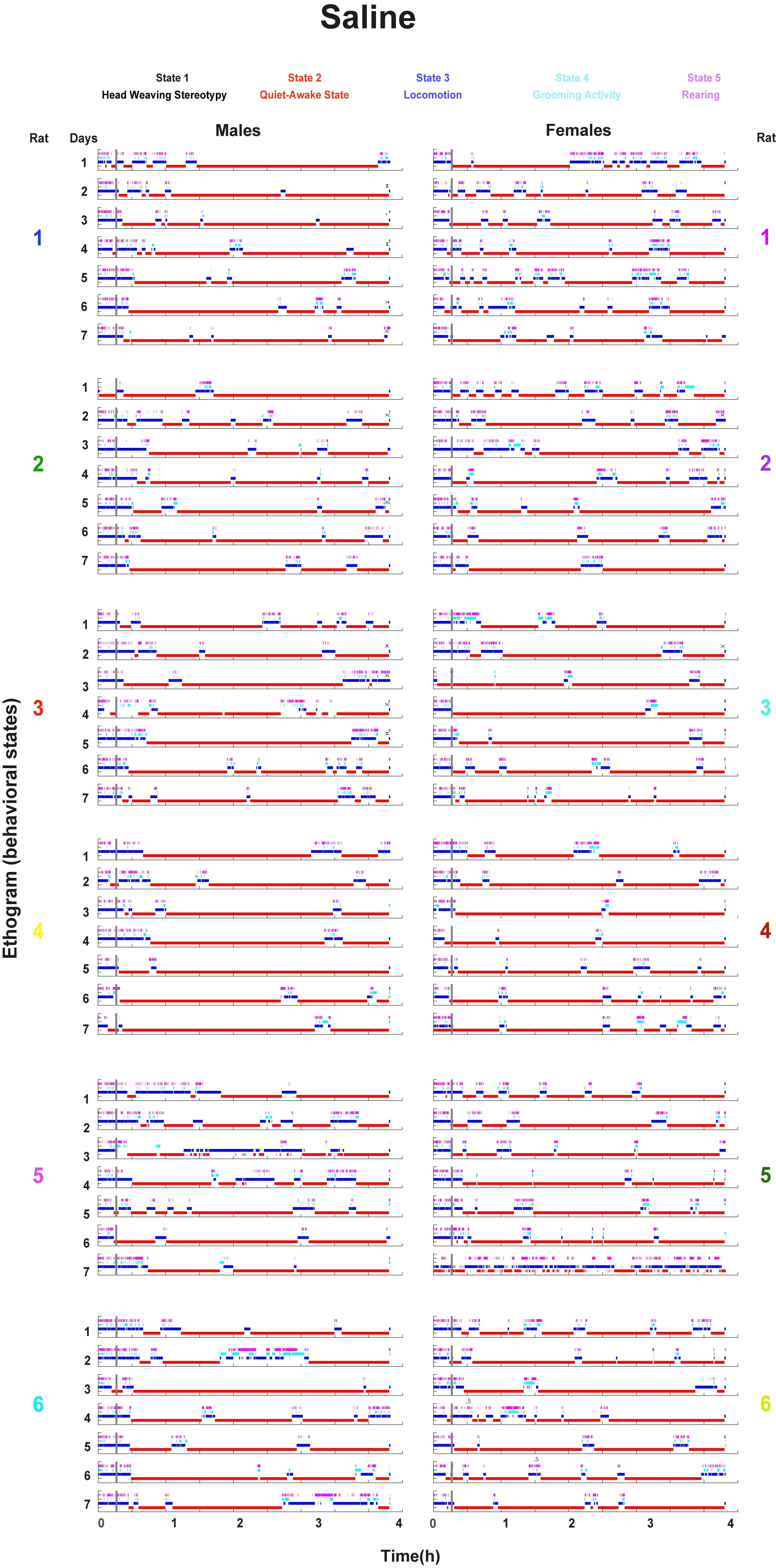

Supplement: S1 Fig — Video frames were analyzed to classify rat behavior over time. Each frame was assigned to one of five mutually exclusive behavioral categories: stereotypy, quiet-awake state, locomotion, grooming, or rearing. For visualization purposes, each behavior was represented by a distinct color: Stereotypy: Black, Quiet-awake state: Red, Locomotion: Blue, Grooming: Cyan, and Rearing: Magenta. The left column displays six panels, each representing one of the six male rats, showing the ethogram for each of the seven treatment days. The right column mirrors the left column, but for the female rats. (TIF) [file pone.0325067.s001.tif]

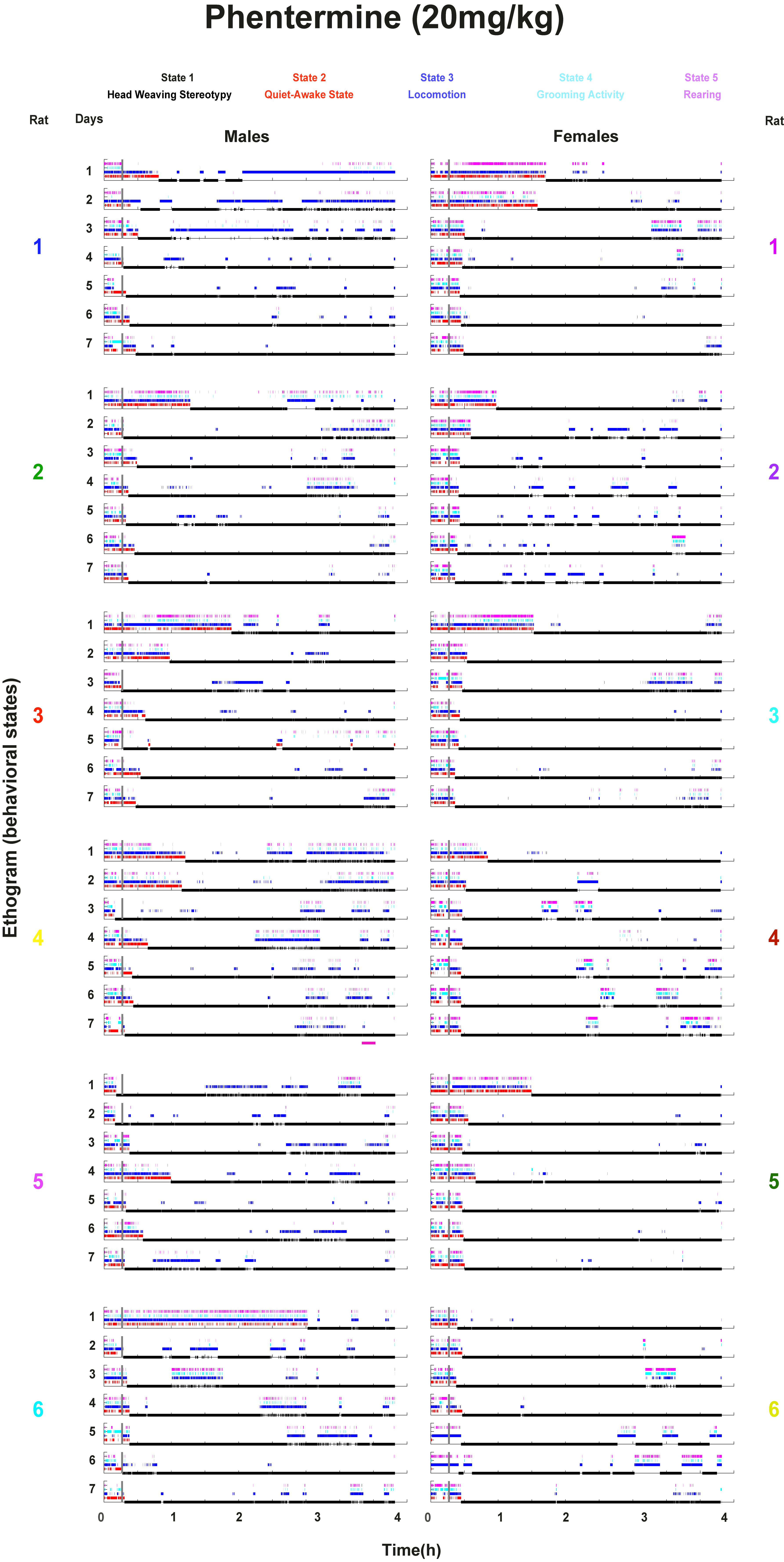

Supplement: S2 Fig — Same as for S1 Fig. For visualization purposes, each behavior was represented by a distinct color: Stereotypy: Black, Quiet-awake state: Red, Locomotion: Blue, Grooming: Cyan, and Rearing: Magenta. The left column displays six panels, each representing one of the six male rats, showing the ethogram for each of the seven days of treatment. The right column mirrors the left column, but for the female rats. (TIF) [file pone.0325067.s002.tif]

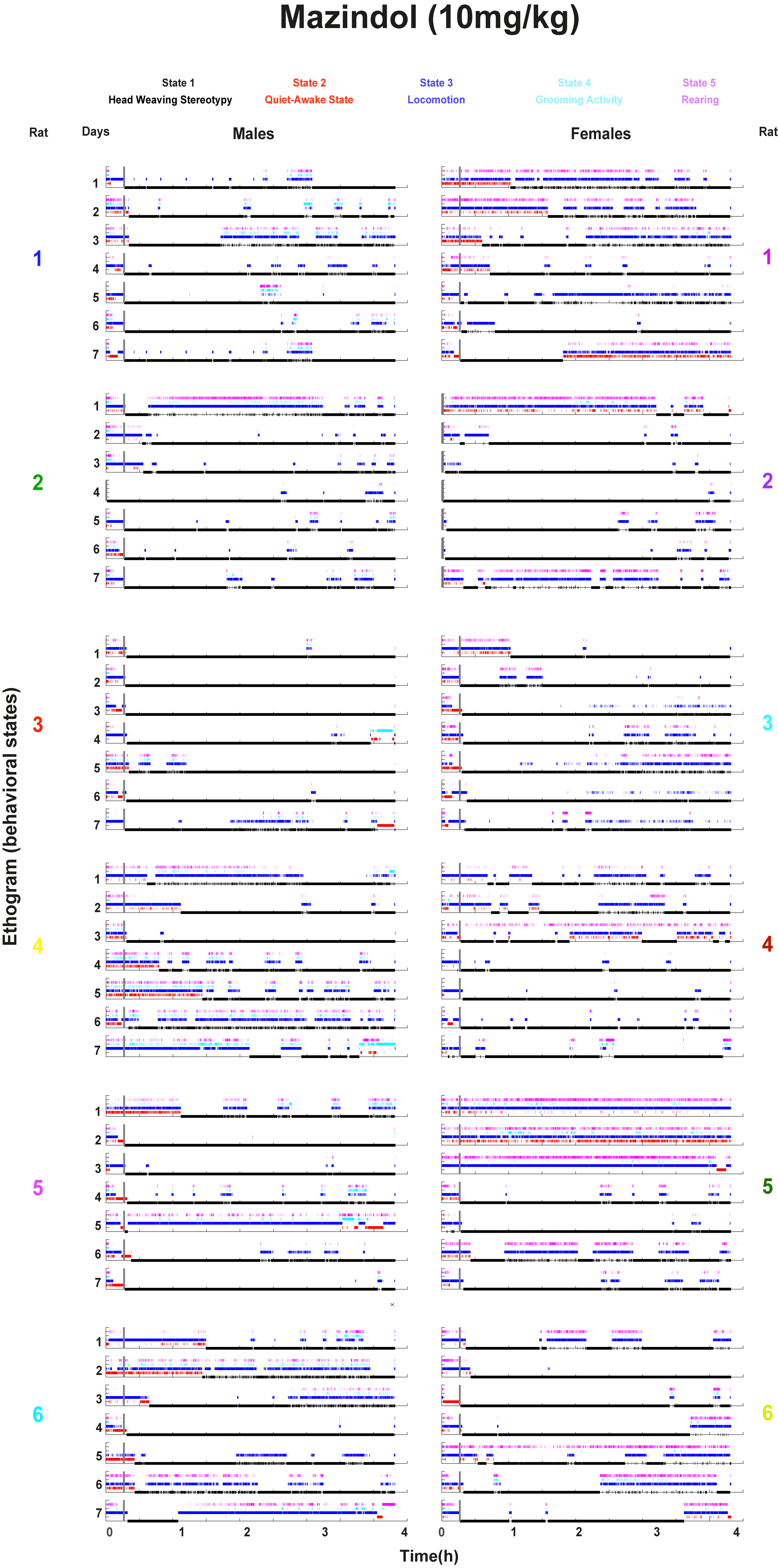

Supplement: S3 Fig — Same as for S1 Fig. For visualization purposes, each behavior was represented by a distinct color: Stereotypy: Black, Quiet-awake state: Red, Locomotion: Blue, Grooming: Cyan, and Rearing: Magenta. The left column displays six panels, each representing one of the six male rats, showing the ethogram for each of the seven days of treatment. The right column mirrors the left column, but for the female rats. (TIF) [file pone.0325067.s003.tif]

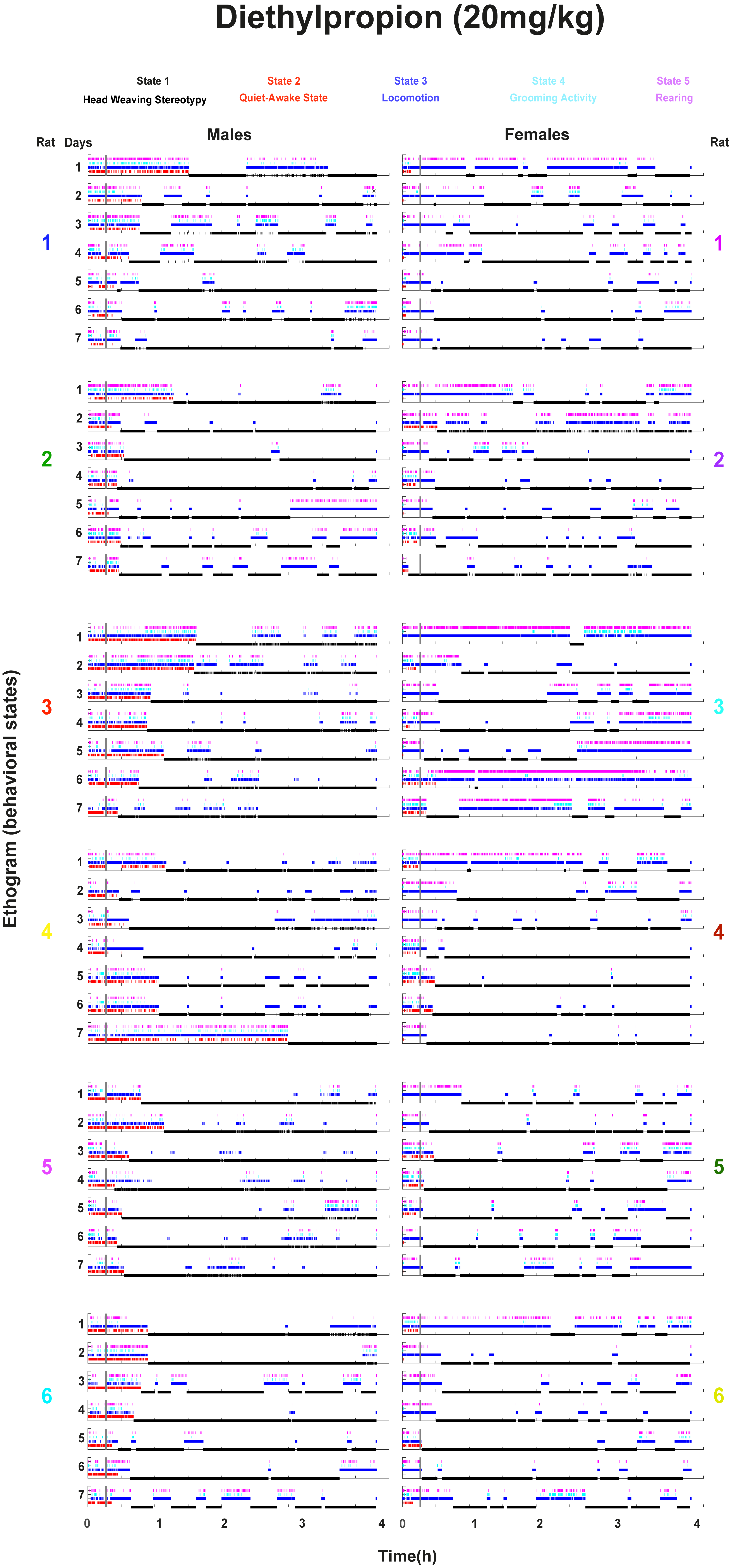

Supplement: S4 Fig — Same as for S1 Fig. For visualization purposes, each behavior was represented by a distinct color: Stereotypy: Black, Quiet-awake state: Red, Locomotion: Blue, Grooming: Cyan, and Rearing: Magenta. The left column displays six panels, each representing one of the six male rats, showing the ethogram for each of the seven days of treatment. The right column mirrors the left column, but for the female rats. (TIF) [file pone.0325067.s004.tif]

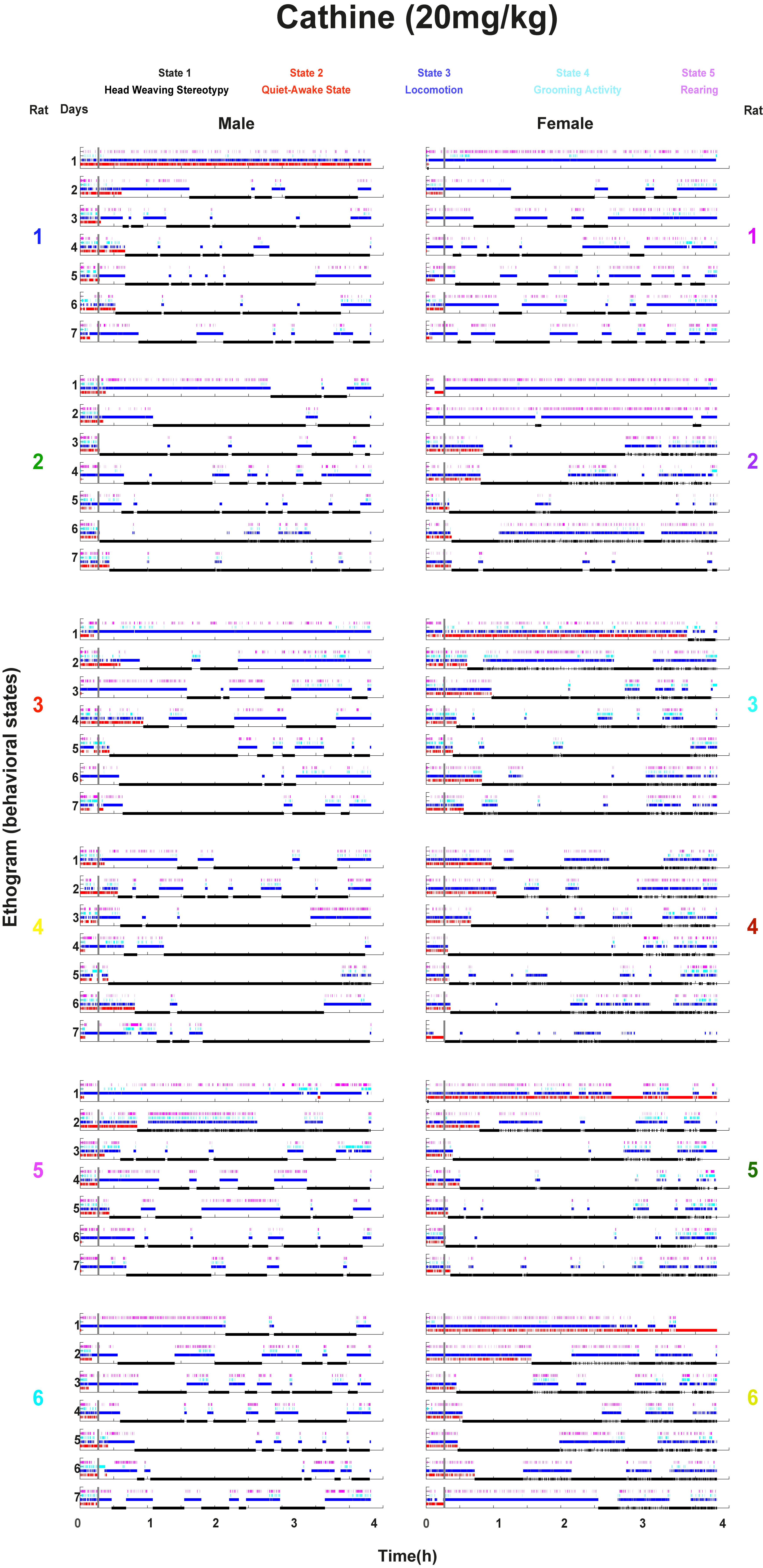

Supplement: S5 Fig — Same as for S1 Fig. For visualization purposes, each behavior was represented by a distinct color: Stereotypy: Black, Quiet-awake state: Red, Locomotion: Blue, Grooming: Cyan, and Rearing: Magenta. The left column displays six panels, each representing one of the six male rats, showing the ethogram for each of the seven days of treatment. The right column mirrors the left column, but for the female rats. (TIF) [file pone.0325067.s005.tif]

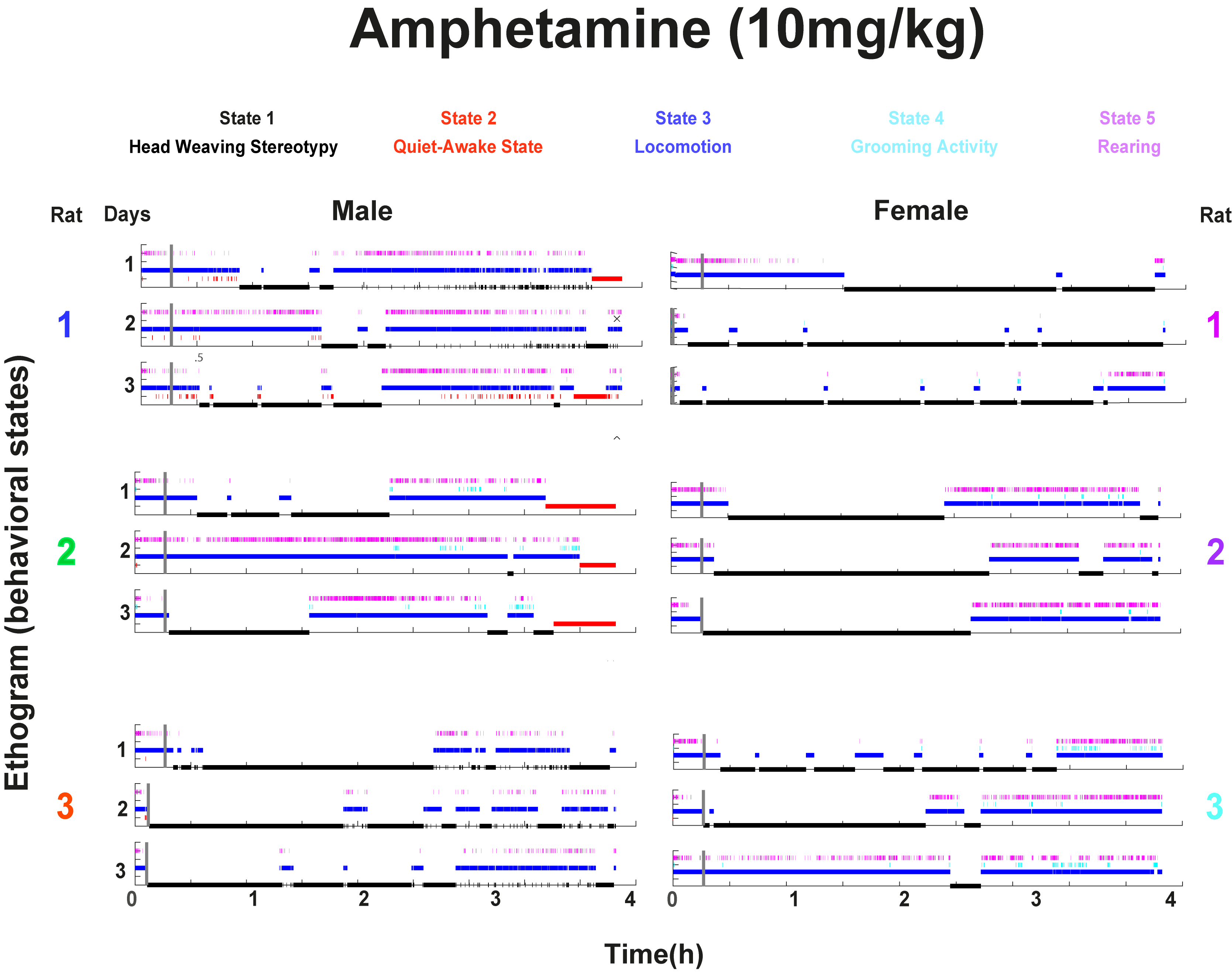

Supplement: S6 Fig — Same as for S1 Fig. For visualization purposes, each behavior was represented by a distinct color: Stereotypy: Black, Quiet-awake state: Red, Locomotion: Blue, Grooming: Cyan, and Rearing: Magenta. The left column displays three panels, each representing one of the three male rats, showing the ethogram for each of the three days of treatment. The right column mirrors the left column, but for the female rats. (TIF) [file pone.0325067.s006.tif]

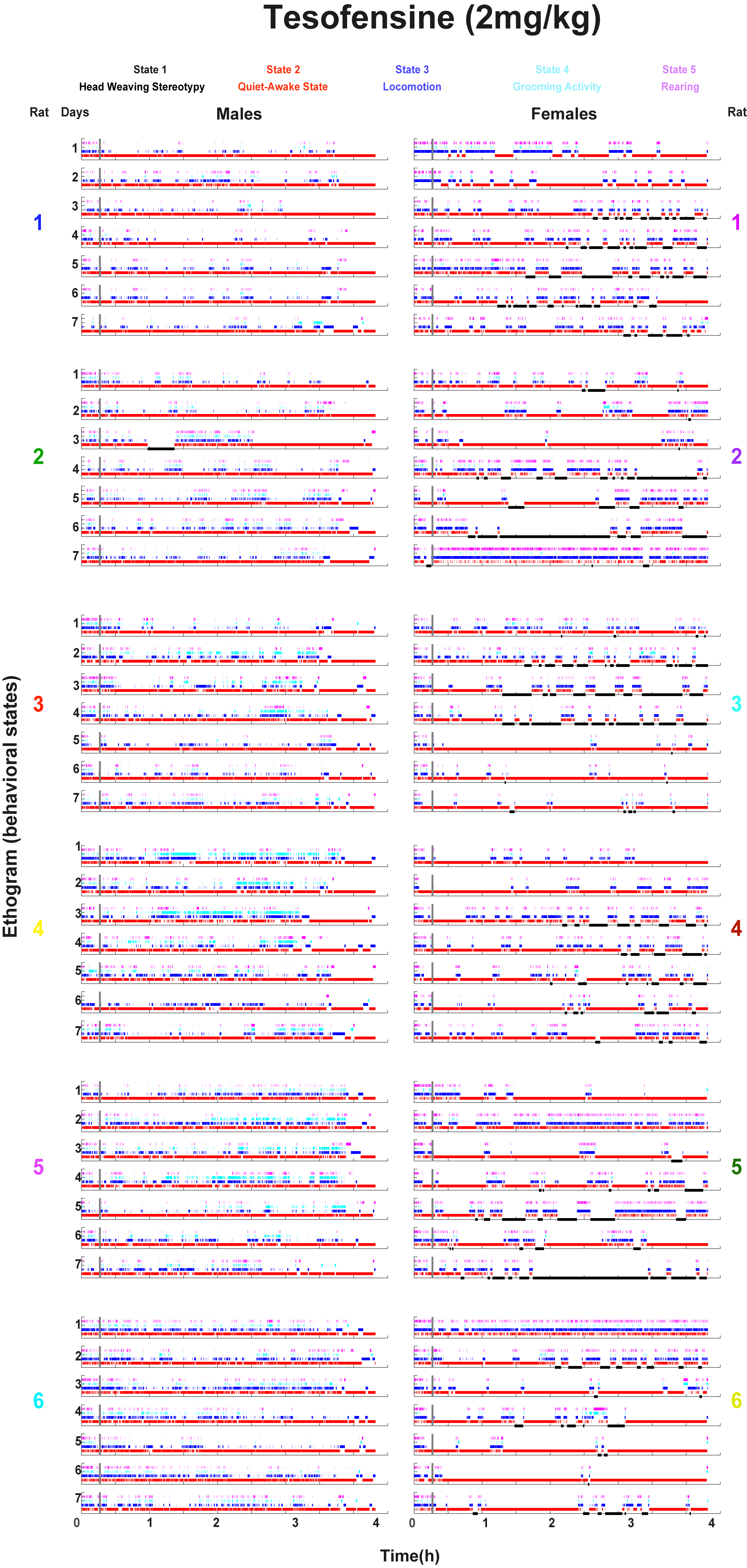

Supplement: S7 Fig — Same as for S1 Fig. For visualization purposes, each behavior was represented by a distinct color: Stereotypy: Black, Quiet-awake state: Red, Locomotion: Blue, Grooming: Cyan, and Rearing: Magenta. The left column displays six panels, each representing one of the six male rats, showing the ethogram for each of the seven days of treatment. The right column mirrors the left column, but for the female rats. (TIF) [file pone.0325067.s007.tif]

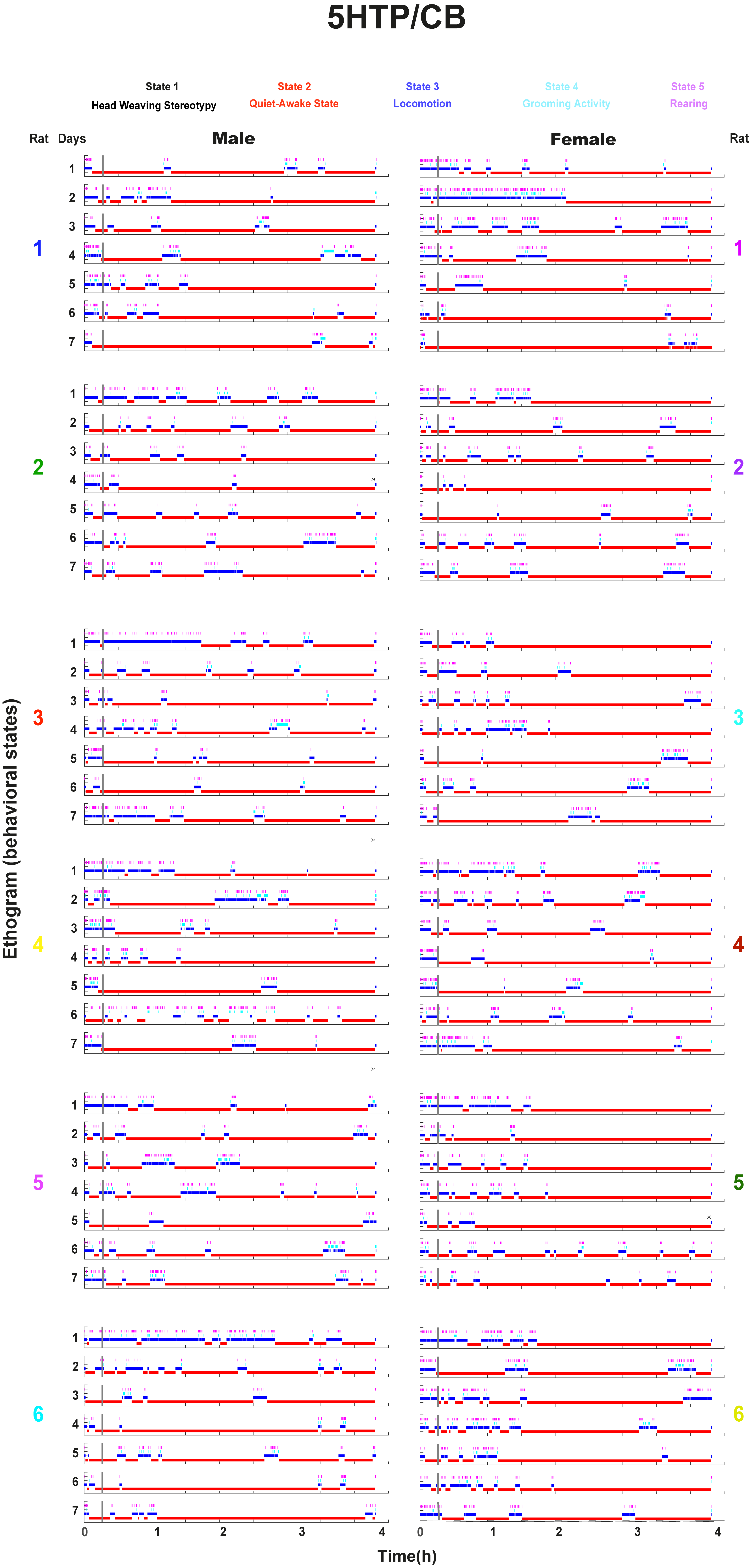

Supplement: S8 Fig — Same as for S1 Fig. For visualization purposes, each behavior was represented by a distinct color: Stereotypy: Black, Quiet-awake state: Red, Locomotion: Blue, Grooming: Cyan, and Rearing: Magenta. The left column displays six panels, each representing one of the six male rats, showing the ethogram for each of the seven days of treatment. The right column mirrors the left column, but for the female rats. (TIF) [file pone.0325067.s008.tif]
